# Laboratory-Confirmed COVID-19 Among Adults Hospitalized with COVID-19–Like Illness with Infection-Induced or mRNA Vaccine-Induced SARS-CoV-2 Immunity — Nine States, January–September 2021

**DOI:** 10.15585/mmwr.mm7044e1

**Published:** 2021-11-05

**Authors:** Catherine H. Bozio, Shaun J. Grannis, Allison L. Naleway, Toan C. Ong, Kristen A. Butterfield, Malini B. DeSilva, Karthik Natarajan, Duck-Hye Yang, Suchitra Rao, Nicola P. Klein, Stephanie A. Irving, Brian E. Dixon, Kristin Dascomb, I-Chia Liao, Sue Reynolds, Charlene McEvoy, Jungmi Han, Sarah E. Reese, Ned Lewis, William F. Fadel, Nancy Grisel, Kempapura Murthy, Jill Ferdinands, Anupam B. Kharbanda, Patrick K. Mitchell, Kristin Goddard, Peter J. Embi, Julie Arndorfer, Chandni Raiyani, Palak Patel, Elizabeth A. Rowley, Bruce Fireman, Nimish R. Valvi, Eric P. Griggs, Matthew E. Levy, Ousseny Zerbo, Rachael M. Porter, Rebecca J. Birch, Lenee Blanton, Sarah W. Ball, Andrea Steffens, Natalie Olson, Jeremiah Williams, Monica Dickerson, Meredith McMorrow, Stephanie J. Schrag, Jennifer R. Verani, Alicia M. Fry, Eduardo Azziz-Baumgartner, Michelle Barron, Manjusha Gaglani, Mark G. Thompson, Edward Stenehjem

**Affiliations:** ^1^CDC COVID-19 Response Team; ^2^Center for Biomedical Informatics, Regenstrief Institute, Indianapolis, Indiana; ^3^Indiana University School of Medicine, Indianapolis, Indiana; ^4^Center for Health Research, Kaiser Permanente Northwest, Portland, Oregon; ^5^Department of Medicine, University of Colorado, Anschutz Medical Campus, Aurora, Colorado; ^6^Westat, Rockville, Maryland; ^7^HealthPartners Institute, Minneapolis, Minnesota; ^8^Department of Biomedical Informatics, Columbia University, New York, New York; ^9^New York Presbyterian Hospital, New York City, New York; ^10^Kaiser Permanente Vaccine Study Center, Kaiser Permanente Northern California, Oakland, California; ^11^Fairbanks School of Public Health, Indiana University, Indianapolis, Indiana; ^12^Division of Infectious Diseases and Clinical Epidemiology, Intermountain Healthcare, Salt Lake City, Utah; ^13^Baylor Scott & White Health, Texas A&M University College of Medicine, Temple, Texas; ^14^Children’s Minnesota, Minneapolis, Minnesota; ^15^Regenstrief Institute, Indianapolis, Indiana.

Previous infection with SARS-CoV-2 (the virus that causes COVID-19) or COVID-19 vaccination can provide immunity and protection from subsequent SARS-CoV-2 infection and illness. CDC used data from the VISION Network* to examine hospitalizations in adults with COVID-19–like illness and compared the odds of receiving a positive SARS-CoV-2 test result, and thus having laboratory-confirmed COVID-19, between unvaccinated patients with a previous SARS-CoV-2 infection occurring 90–179 days before COVID-19–like illness hospitalization, and patients who were fully vaccinated with an mRNA COVID-19 vaccine 90–179 days before hospitalization with no previous documented SARS-CoV-2 infection. Hospitalized adults aged ≥18 years with COVID-19–like illness were included if they had received testing at least twice: once associated with a COVID-19–like illness hospitalization during January–September 2021 and at least once earlier (since February 1, 2020, and ≥14 days before that hospitalization). Among COVID-19–like illness hospitalizations in persons whose previous infection or vaccination occurred 90–179 days earlier, the odds of laboratory-confirmed COVID-19 (adjusted for sociodemographic and health characteristics) among unvaccinated, previously infected adults were higher than the odds among fully vaccinated recipients of an mRNA COVID-19 vaccine with no previous documented infection (adjusted odds ratio [aOR] = 5.49; 95% confidence interval [CI] = 2.75–10.99). These findings suggest that among hospitalized adults with COVID-19–like illness whose previous infection or vaccination occurred 90–179 days earlier, vaccine-induced immunity was more protective than infection-induced immunity against laboratory-confirmed COVID-19. All eligible persons should be vaccinated against COVID-19 as soon as possible, including unvaccinated persons previously infected with SARS-CoV-2.

To compare the early protection against COVID-19 conferred by SARS-CoV-2 infection and by receipt of mRNA COVID-19 vaccines (i.e., 90–179 days after infection or vaccination), the VISION Network collected data from 187 hospitals across nine states during January–September 2021 (*1*). Eligible hospitalizations were defined as those among adults aged ≥18 years who had received SARS-CoV-2 molecular testing (from 14 days before to 72 hours after admission) and had a COVID-19–like illness discharge diagnosis^†^ during January–September 2021. Eligible patients had also been tested at least once since February 1, 2020. To limit the analysis to patients with access to SARS-CoV-2 testing before hospitalization, patients who did not receive SARS-CoV-2 testing ≥14 days before hospitalization were excluded.

Two exposure groups were defined based on COVID-19 vaccination status and previous SARS-CoV-2 infection. Vaccination status was documented in electronic health records and immunization registries. Previous infection was ascertained based on SARS-CoV-2 testing from rapid antigen tests or molecular assays (e.g., real-time reverse transcription–polymerase chain reaction) performed before mRNA vaccination and ≥14 days before admission; testing performed after February 2020 was primarily within network partners’ medical facilities. Adults were considered unvaccinated with a previous SARS-CoV-2 infection if no COVID-19 vaccine doses were received and if the most recent positive SARS-CoV-2 test result occurred ≥90 days before hospitalization. Adults were considered fully vaccinated with an mRNA COVID-19 vaccine with no previous documented infection if the second dose of Pfizer-BioNTech (BNT162b2) or Moderna (mRNA-1273) mRNA vaccine was received ≥14 days before the index test date^§^ and if they had been tested since February 1, 2020, and had no positive test results ≥14 days before hospitalization. Patients were excluded if they had received 1 mRNA vaccine dose only, received the second dose <14 days before index test date, or received the Janssen (Johnson & Johnson [Ad26.COV2]) vaccine (because of sparse data). To reduce the chance that the hospitalization was related to an ongoing SARS-CoV-2 infection, patients were also excluded from the previous infection group if their most recent previous positive test result occurred 14–89 days before hospitalization.^¶^

The outcome of laboratory-confirmed COVID-19 was defined as COVID-19–like illness and a positive SARS-CoV-2 result from molecular testing. Among patients hospitalized with COVID-19–like illness whose previous infection or completion of vaccination occurred 90–179 days earlier, the odds of laboratory-confirmed COVID-19 were compared between previously infected persons and fully vaccinated mRNA COVID-19 vaccine recipients. aORs and 95% CIs were calculated using multivariable logistic regression, adjusted for age, geographic region, calendar time (days from January 1 to hospitalization), and local virus circulation, and weighted based on propensity to be in the vaccinated category (*1*,*2*). Established methods were used to calculate weights to account for differences in sociodemographic and health characteristics between groups (*3*). Separate weights were calculated for each model. aORs were stratified by mRNA vaccine product and age group.

Three secondary analyses were also conducted. First, the impact of whether and how the time interval since previous infection or full vaccination was adjusted was examined. Specifically, any time since either previous infection or completion of vaccination was considered. Then, previously infected patients were limited to those with more recent infections (i.e., 90–225 days before hospitalization [the lowest two tertiles of number of days since infection]), and fully vaccinated patients were limited to those with the longest interval since completion of vaccination (i.e., receipt of second mRNA vaccine dose 45–213 days before hospitalization [the highest two tertiles of number of days since vaccination]). Then, number of days since previous infection or completion of vaccination, rather than calendar time, was adjusted in the model. For the next secondary analysis, aORs for hospitalizations that occurred before and during SARS-CoV-2 B.1.617.2 (Delta) variant predominance (June–September 2021) were compared, beginning on the date the Delta variant accounted for >50% of sequenced isolates in each medical facility’s state (*2*). Finally, effect modification was assessed by mRNA vaccine product or by age group; p-values <0.2 were considered indicative of a statistically significant difference in aOR by product or age, similar to previous modeling studies of effect modification (*4*). All analyses were conducted using SAS (version 9.4; SAS Institute) and R (version 4.0.2; R Foundation). This study was reviewed and approved by Westat, Inc. institutional review board.**

During January 1–September 2, 2021, a total of 201,269 hospitalizations for COVID-19–like illness were identified; 139,655 (69.4%) patients were hospitalized after COVID-19 vaccines were generally available to persons in their age group within their geographic region. Molecular testing for SARS-CoV-2 was performed for 94,264 (67.5%) patients with COVID-19–like illness hospitalizations. Among these patients, 7,348 (7.8%) had at least one other SARS-CoV-2 test result ≥14 days before hospitalization and met criteria for either of the two exposure categories: 1,020 hospitalizations were among previously infected and unvaccinated persons, and 6,328 were among fully vaccinated and previously uninfected patients (Table 1).

**TABLE 1:** Characteristics of COVID-19–like illness hospitalizations* among unvaccinated adults with a SARS-CoV-2 infection occurring 90–179 days before the index test date^†^ and among adults who were fully vaccinated^§^ 90–179 days before the index test date^†^ without a previous documented SARS-CoV-2 infection — nine states,**^¶^** January–September 2021

Characteristic	No. (column %)		Standardized mean or proportion difference**
	Unvaccinated with previous SARS-CoV-2 infection	Fully vaccinated^§^ without previous documented infection	
**All hospitalizations with COVID-19–like illness**	**1,020 (100)**	**6,328 (100)**	NA
**SARS-CoV-2 test result associated with COVID-19–like illness hospitalization**			
Positive	89 (9)	324 (5)	0.14
Negative	931 (91)	6,004 (95)	
**Sex**			
Male	405 (40)	2,905 (46)	0.13
Female	615 (60)	3,423 (54)	
**Age group, yrs**			
18–49	313 (31)	560 (9)	0.74
50–64	243 (24)	865 (14)	
65–74	207 (20)	1,757 (28)	
75–84	177 (17)	2,018 (32)	
≥85	80 (8)	1,128 (18)	
**Race, irrespective of ethnicity**			
White	647 (63)	4,356 (69)	0.24
Black	100 (10)	452 (7)	
Other**^††^**	71 (7)	686 (11)	
Unknown	202 (20)	834 (13)	
**Ethnicity, irrespective of race**			
Hispanic	189 (19)	756 (12)	0.20
Non-Hispanic	695 (68)	4,458 (70)	
Unknown	136 (13)	1,114 (18)	
**Month of index test date^†^**			
January	11 (1)	0 (—)	2.10
February	41 (4)	0 (—)	
March	114 (11)	0 (—)	
April	245 (24)	6 (0)	
May	294 (29)	235 (4)	
June	184 (18)	1,300 (21)	
July	99 (10)	2,731 (43)	
August	31 (3)	2,049 (32)	
September	1 (0)	7 (0)	
**Site**			
Columbia University	53 (5)	238 (4)	0.73
HealthPartners	22 (2)	94 (1)	
Intermountain Healthcare	117 (11)	454 (7)	
Kaiser Permanente Northern California	254 (25)	3,614 (57)	
Kaiser Permanente Northwest	30 (3)	250 (4)	
Regenstrief Institute	390 (38)	1,145 (18)	
University of Colorado	154 (15)	533 (8)	
**Time since either previous SARS-CoV-2 infection or full mRNA vaccination until COVID-19–like illness index test date, days**			
90–119	367 (36)	3,325 (53)	0.42
120–149	353 (35)	2,101 (33)	
150–179	300 (29)	902 (14)	
**COVID-19 vaccination status**			
Unvaccinated	1,020 (100)	0 (—)	NA
Pfizer-BioNTech (BNT162b2)	0 (—)	3,736 (59)	
Moderna (mRNA-1273)	0 (—)	2,592 (41)	

**Abbreviation:** NA = not applicable.

* Medical events with a discharge code consistent with COVID-19–like illness were included. COVID-19–like illness diagnoses included acute respiratory illness (e.g., COVID-19, respiratory failure, or pneumonia) or related signs or symptoms (cough, fever, dyspnea, vomiting, or diarrhea) using diagnosis codes from the *International Classification of Diseases, Ninth Revision* and *International Classification of Diseases, Tenth Revision*. Clinician-ordered molecular assays (e.g., real-time reverse transcription–polymerase chain reaction) for SARS-CoV-2 occurring ≤14 days before to <72 hours after hospital admission were included.

^†^ Index test date was defined as the date of respiratory specimen collection associated with the most recent positive or negative SARS-CoV-2 test result before the hospitalization or the hospitalization date if testing only occurred after the admission.

^§^ Full vaccination was defined as receipt of the second dose of Pfizer-BioNTech or Moderna mRNA vaccine ≥14 days before the index test date.

^¶^ Partners contributing hospitalizations were in California, Colorado, Indiana, Minnesota and Wisconsin, Oregon and Washington, Utah, and New York.

** In comparing characteristics between unvaccinated adults with a previous infection and fully vaccinated adults without a previous documented infection, a standardized mean or proportion difference >0.2 was considered noteworthy. After balancing characteristics that differed between the two comparison groups, the standardized mean or proportion differences were ≤0.06.

^††^ Other race includes Asian, Hawaiian or Other Pacific islander, American Indian or Alaskan Native, Other not listed, and multiple races.

Laboratory-confirmed SARS-CoV-2 infection was identified among 324 (5.1%) of 6,328 fully vaccinated persons and among 89 of 1,020 (8.7%) unvaccinated, previously infected persons. A higher proportion of previously infected than vaccinated patients were aged 18–49 years (31% versus 9%), Black (10% versus 7%), and Hispanic (19% versus 12%).

Among COVID-19–like illness hospitalizations in persons whose previous infection or vaccination occurred 90–179 days earlier, the odds of laboratory-confirmed COVID-19 were higher among previously infected, unvaccinated patients than among fully vaccinated patients (aOR = 5.49; 95% CI = 2.75–10.99) (Table 2). In secondary analyses, the aORs that examined the impact of whether and how time since infection or vaccination was adjusted and that stratified hospitalizations before and during Delta variant predominance were all similar to the primary aOR estimate. For product- and age group–specific estimates, sparse data limited the precision of these aORs. However, an assessment of effect modification indicated the aOR of laboratory-confirmed COVID-19 was higher for previously infected patients compared with patients vaccinated with Moderna (aOR = 7.30) than compared with patients vaccinated with Pfizer-BioNTech (aOR = 5.11) during January–September (p = 0.02). Similarly, the interaction term for exposure group by age indicated that the aOR was higher for patients aged ≥65 years (aOR = 19.57) than for those aged 18–64 years (aOR = 2.57) (interaction term, p = 0.05).

**TABLE 2:** Adjusted odds ratios* of laboratory-confirmed COVID-19 among hospitalizations in adults with COVID-19–like illness comparing unvaccinated adults with a SARS-CoV-2 infection occurring 90–179 days before the index test date and adults who were fully vaccinated 90–179 days before the index test date without a previous documented SARS-CoV-2 infection — nine states, January–September 2021

Outcome	Total no.	No. (row %) of SARS-CoV-2 positive test results	Adjusted odds ratio (95% CI)
**All adults (aged ≥18 years), any COVID-19 mRNA vaccine**			
**Any mRNA vaccine**			
Fully vaccinated^†^ without previous documented infection	6,328	324 (5.1)	Ref
Unvaccinated with a previous SARS-CoV-2 infection	1,020	89 (8.7)	5.49 (2.75–10.99)
**Any mRNA vaccine, no restriction of time since previous infection or completion of vaccination**			
Fully vaccinated^†^ without previous documented infection (range of time since vaccination = 0–213 days before hospitalization)	18,397	542 (3.0)	Ref
Unvaccinated with a previous SARS-CoV-2 infection (range of time since previous infection = 90–494 days before hospitalization)	2,085	130 (6.2)	2.75 (1.90–3.98)
**Any mRNA vaccine, examining the potential influence of time since previous infection or completion of vaccination**			
Fully vaccinated^†^ without previous documented infection, limited to those with longest period since vaccination (range of time since vaccination = 45–213 days before hospitalization)	12,231	458 (3.7)	Ref
Unvaccinated with a previous SARS-CoV-2 infection, limited to those with more recent infections (range of time since previous infection = 90–225 days before hospitalization)	1,389	107 (7.7)	3.98 (2.49–6.35)
**Any mRNA vaccine, adjusting for time since previous infection or completion of vaccination in model**			
Fully vaccinated^†^ without previous documented infection	6,328	324 (5.1)	Ref
Unvaccinated with a previous SARS-CoV-2 infection	1,020	89 (8.7)	3.22 (1.68–6.20)
**By time relative to SARS-CoV-2 B.1.617.2 (Delta) variant predominance**			
**Before Delta predominance (January–June 2021)**			
Fully vaccinated^†^ without previous documented infection	1,115	18 (1.6)	Ref
Unvaccinated with a previous SARS-CoV-2 infection	831	70 (8.4)	6.11 (2.83–13.16)
During Delta predominance (June–September 2021)**			
Fully vaccinated^†^ without previous documented infection	5,213	306 (5.9)	Ref
Unvaccinated with a previous SARS-CoV-2 infection	189	19 (10.1)	7.55 (3.45–16.52)
**By mRNA vaccine product^§^**			
**Pfizer-BioNTech (BNT162b2)**			
Fully vaccinated^†^ without previous documented infection	3,736	215 (5.8)	Ref
Unvaccinated with a previous SARS-CoV-2 infection	1,020	89 (8.7)	5.11 (2.53–10.29)
**Moderna (mRNA-1273)**			
Fully vaccinated^†^ without previous documented infection	2,592	109 (4.2)	Ref
Unvaccinated with a previous SARS-CoV-2 infection	1,020	89 (8.7)	7.30 (3.40–15.60)
**By age group, yrs^¶^**			
**18–64**			
Fully vaccinated^†^ without previous documented infection	1,425	71 (5.0)	Ref
Unvaccinated with a previous SARS-CoV-2 infection	556	49 (8.8)	2.57 (1.42–4.65)
**≥65**			
Fully vaccinated^†^ without previous documented infection	4,903	253 (5.2)	Ref
Unvaccinated with a previous SARS-CoV-2 infection	464	40 (8.6)	19.57 (8.34–45.91)

**Abbreviations:** CI = confidence interval; ref = referent group.

* Odds ratios were adjusted for age, geographic region, calendar time (days since January 1, 2021), and local virus circulation (percentage of SARS-CoV-2 positive results from testing within the counties surrounding the facility on the date of the hospitalization) and balanced using inverse weights on characteristics that differed between the two groups (calculated separately for each odds ratio model) using facility characteristics, sociodemographic characteristics, and underlying medical conditions. Cardiovascular disease was also adjusted in the main model and in the model for Pfizer-BioNTech. Any likely immunosuppression was also included in the model for Moderna. Neuromuscular and respiratory conditions were also adjusted in the model for adults aged ≥65 years. Number of days since previous infection or completion of vaccination, instead of calendar time, was adjusted in the model within the stated secondary analysis.

^†^ Full vaccination was defined as receipt of the second dose of Pfizer-BioNTech or Moderna mRNA vaccine ≥14 days before the index test date.

^§^ P-value from assessment of effect modification by mRNA product was 0.02.

^¶^ P-value for interaction term for exposure group by age group was 0.05.

** SARS-CoV-2 B.1.617.2 (Delta) variant predominance began on the date the Delta variant accounted for >50% of sequenced isolates in each medical facility’s state. https://doi.org/10.15585/mmwr.mm7037e2

## Discussion

In this multistate analysis of hospitalizations for COVID-19–like illness among adults aged ≥18 years during January–September 2021 whose previous infection or vaccination occurred 90–179 days earlier, the adjusted odds of laboratory-confirmed COVID-19 were higher among unvaccinated and previously infected patients than among those who were fully vaccinated with 2 doses of an mRNA COVID-19 vaccine without previous documentation of a SARS-CoV-2 infection. Secondary analyses that did not adjust for time since infection or vaccination or adjusted time since infection or vaccination differently as well as before and during Delta variant predominance produced similar results. These findings are consistent with evidence that neutralizing antibody titers after receipt of 2 doses of mRNA COVID-19 vaccine are high (*5*,*6*); however, these findings differ from those of a retrospective records-based cohort study in Israel,^††^ which did not find higher protection for vaccinated adults compared with those with previous infection during a period of Delta variant circulation. This variation is possibly related to differences in the outcome of interest and restrictions on the timing of vaccination. The Israeli cohort study assessed any positive SARS-CoV-2 test result, whereas this study examined laboratory-confirmed COVID-19 among hospitalized patients. The Israeli cohort study also only examined vaccinations that had occurred 6 months earlier, so the benefit of more recent vaccination was not examined. This report focused on the early protection from infection-induced and vaccine-induced immunity, though it is possible that estimates could be affected by time. Understanding infection-induced and vaccine-induced immunity over time is important, particularly for future studies to consider. 

In this study, the benefit of vaccination compared with infection without vaccination appeared to be higher for recipients of Moderna than Pfizer-BioNTech vaccine, which is consistent with a recent study that found higher vaccine effectiveness against COVID-19 hospitalizations for Moderna vaccine recipients than for Pfizer-BioNTech vaccine recipients (*7*). In this study, the protective effect of vaccination also trended higher for adults aged ≥65 years than for those aged 18–64 years. However, considering the limited data by both product type and age, additional research is needed on the relative protection of vaccination versus infection without vaccination across demographic groups and vaccine products, as well as vaccination in previously infected persons.

The findings in this report are subject to at least seven limitations. First, although this analysis was designed to compare two groups with different sources of immunity, patients might have been misclassified. If SARS-CoV-2 testing occurred outside of network partners' medical facilities or if vaccinated persons are less likely to seek testing, some positive SARS-CoV-2 test results might have been missed and thus some patients classified as vaccinated and previously uninfected might also have been infected. In addition, despite the high specificity of COVID-19 vaccination status from these data sources, misclassification is possible. Second, the aOR could not be further stratified by time since infection or vaccination because of sparse data and limited ability to control for residual confounding that could be magnified within shorter intervals. The aOR that did not adjust for time might also be subject to residual confounding, particularly related to waning of both types of immunity. Third, selection bias might be possible if vaccination status influences likelihood of testing and if previous infection influences the likelihood of vaccination. Previous work from the VISION network did not identify systematic bias in testing by vaccination status, based on data through May 2021 (*1*). Fourth, residual confounding might exist because the study did not measure or adjust for behavioral differences between the comparison groups that could modify the risk of the outcome. Fifth, these results might not be generalizable to nonhospitalized patients who have different access to medical care or different health care–seeking behaviors, particularly outside of the nine states covered. Sixth, the statistical model incorporated the use of a weighted propensity score method which is subject to biases in estimates or standard errors if the propensity score model is misspecified. Numerous techniques were used to reduce potential suboptimal specification of the model, including but not limited to including a large set of covariates for machine learning estimation of propensity scores, including covariates in both regression and propensity models, ensuring large sample sizes and checking stability of weights, and conducting secondary analyses to assess robustness of results. Finally, the study assessed COVID-19 mRNA vaccines only; findings should not be generalized to the Janssen vaccine.

In this U.S.-based epidemiologic analysis of patients hospitalized with COVID-19–like illness whose previous infection or vaccination occurred 90–179 days earlier, vaccine-induced immunity was more protective than infection-induced immunity against laboratory-confirmed COVID-19, including during a period of Delta variant predominance. All eligible persons should be vaccinated against COVID-19 as soon as possible, including unvaccinated persons previously infected with SARS-CoV-2.

**Summary**

**What is already known about this topic?**

Previous infection with SARS-CoV-2 or COVID-19 vaccination can provide immunity and protection against subsequent SARS-CoV-2 infection and illness.

**What is added by this report?**

Among COVID-19–like illness hospitalizations among adults aged ≥18 years whose previous infection or vaccination occurred 90–179 days earlier, the adjusted odds of laboratory-confirmed COVID-19 among unvaccinated adults with previous SARS-CoV-2 infection were 5.49-fold higher than the odds among fully vaccinated recipients of an mRNA COVID-19 vaccine who had no previous documented infection (95% confidence interval = 2.75–10.99).

**What are the implications for public health practice?**

All eligible persons should be vaccinated against COVID-19 as soon as possible, including unvaccinated persons previously infected with SARS-CoV-2.

## Footnotes

* Funded by CDC, the VISION Network includes Columbia University Irving Medical Center (New York), HealthPartners (Minnesota and Wisconsin), Intermountain Healthcare (Utah), Kaiser Permanente Northern California (California), Kaiser Permanente Northwest (Oregon and Washington), Regenstrief Institute (Indiana), and University of Colorado (Colorado).

† Medical events with a discharge code consistent with COVID-19–like illness were included. COVID-19–like illness diagnoses included acute respiratory illness (e.g., COVID-19, respiratory failure, or pneumonia) or related signs or symptoms (cough, fever, dyspnea, vomiting, or diarrhea) using diagnosis codes from the *International Classification of Diseases, Ninth Revision* and *International Classification of Diseases, Tenth Revision*.

§ Index test date was defined as the date of respiratory specimen collection associated with the most recent positive or negative SARS-CoV-2 test result before the hospitalization or the hospitalization date if testing only occurred after admission.

¶
https://www.cdc.gov/coronavirus/2019-ncov/php/invest-criteria.html

** 45 C.F.R. part 46; 21 C.F.R. part 56.

††
https://www.medrxiv.org/content/10.1101/2021.08.24.21262415v1
